# MAVMET trial: maraviroc and/or metformin for metabolic dysfunction associated fatty liver disease in adults with suppressed HIV

**DOI:** 10.1097/QAD.0000000000003947

**Published:** 2024-06-25

**Authors:** Leanne McCabe, James E. Burns, Arash Latifoltojar, Frank A. Post, Julie Fox, Erica Pool, Anele Waters, Beatriz Santana, Lucy Garvey, Margaret Johnson, Ian McGuinness, Manil Chouhan, Jonathan Edwards, Anna L. Goodman, Graham Cooke, Claire Murphy, Yolanda Collaco-Moraes, Helen Webb, Adam Gregory, Fatima Mohamed, Mary Rauchenberger, Stephen D. Ryder, Chris Sandford, Jason V. Baker, Brian Angus, Christoph Boesecke, Chloe Orkin, Shonit Punwani, Andrew Clark, Richard Gilson, David Dunn, Sarah L. Pett

**Affiliations:** a Medical Research Council Clinical Trials Unit at UCL, Institute for Clinical Trials and Methodology, University College London (UCL); b Institute for Global Health, UCL; c Mortimer Market Centre, Central and Northwest London NHS Foundation Trust; d UCL Centre for Medical Imaging, UCL; e King's College Hospital NHS Foundation Trust; f King's College London; g Guy's and St Thomas’ NHS Foundation Trust; h Imperial College Healthcare NHS Trust; i Royal Free London NHS Foundation Trust, London; j University of Oxford, Oxford; k NIHR Nottingham Biomedical Research Centre, Nottingham University Hospitals NHS Trust and The University of Nottingham, Nottingham, UK; l Hennepin Healthcare Research Institute, Minneapolis, Minnesota, USA; m University of Bonn, Bonn, Germany; n Royal London Hospital, Barts Health NHS Trust; o Queen Mary University of London; p ViiV Healthcare, London, UK.

**Keywords:** antiretroviral therapy, HIV, maraviroc, metabolic dysfunction associated fatty liver disease, metformin

## Abstract

**Objective:**

Metabolic dysfunction associated fatty liver disease (MAFLD) is over-represented in people with HIV (PWH). Maraviroc (MVC) and/or metformin (MET) may reduce MAFLD by influencing inflammatory pathways and fatty acid metabolism.

**Design:**

Open-label, 48-week randomized trial with a 2 x 2 factorial design.

**Setting:**

Multicenter HIV clinics.

**Participants:**

Nondiabetic, virologically suppressed PLWH, aged at least 35 years, with confirmed/suspected MAFLD (≥1 biochemical/anthropometric/radiological/histological features).

**Intervention:**

Adjunctive MVC; MET; MVC+MET vs. antiretroviral therapy (ART) alone.

**Primary outcome:**

Change in liver fat fraction (LFF) between baseline and week-48 using magnetic resonance proton density fat fraction (MR PDFF).

**Results:**

Six sites enrolled 90 participants (93% male; 81% white; median age 52 [interquartile range, IQR 47–57] years) between March 19, 2018, and November 11, 2019. Seventy percent had imaging/biopsy and at least one 1 MAFLD criteria. The analysis included 82/90 with week-0 and week-48 scans. Median baseline MR PDFF was 8.9 (4.6–17.1); 40, 38, 8, and 14% had grade zero, one, two, and three steatosis, respectively. Mean LFF increased slightly between baseline and follow-up scans: 2.22% MVC, 1.26% MET, 0.81% MVC+MET, and 1.39% ART alone. Prolonged intervention exposure (delayed week-48 scans) exhibited greater increases in MR PDFF (estimated difference 4.23% [95% confidence interval, 95% CI 2.97–5.48], *P* < 0.001). There were no differences in predicted change for any intervention compared to ART alone: MVC (-0.42% [95% CI -1.53 to 0.68, *P* = 0.45]), MET (-0.62 [-1.81 to 0.56, *P* = 0.30]), and MVC+MET (-1.04 [-2.74 to 0.65, *P* = 0.23]). Steatosis grade remained unchanged in 55% and increased in 24%.

**Conclusion:**

Baseline levels of liver fat were lower than predicted. Contrary to our hypothesis, neither MVC, MET, or the combination significantly reduced liver fat as measured by MRPDFF compared to ART alone.

## Introduction

Increasingly, people with HIV (PWH, HIV) have a life expectancy comparable to the general population if taking effective antiretroviral therapy (ART). Consequently, the sequelae of aging including multimorbidity, have become pertinent issues. Metabolic dysfunction associated fatty liver disease (MAFLD, previously known as nonalcoholic fatty liver disease [NALFD]) is one condition that has become increasingly prevalent. MAFLD is a spectrum of disease progressing from hepatic steatosis to metabolic steatohepatitis (MASH), and ultimately, fibrosis that predisposes to end-stage liver disease and hepatocellular carcinoma. It is currently the most common global cause of chronic liver disease [1]. Previous pooled prevalence estimates observed nearly 35% of PWH have NAFLD (now MAFLD) on imaging [2], although recent European HIV cohorts report prevalences more than 60% [3].

One challenge with MAFLD is determining a noninvasive method of diagnosis to avoid liver biopsy, which in itself is flawed, as liver fat distribution is not homogeneous. Traditionally, a liver fat content more than 50 mg/g (5% by wet weight) is diagnostic of hepatic steatosis [4,5] and ^1^H magnetic resonance spectroscopy (MRS) is the gold standard validated against liver biopsies. However, more modern techniques, notably magnetic resonance proton density fat fraction (MR PDFF), have been validated against MRS, histology, or both (text, Supplemental Digital Content 21, radiology methodology references), and have shown strong correlation. MR PDFF is also felt to be potentially more sensitive as longitudinal studies have observed MR PDFF changes correlated with liver enzymes and metabolic markers whilst histological assessment remained static [6–8]. Another advantage of MR PDFF is the ability to assess extra-hepatic fat including pancreatic fat fraction [9], also linked to the development of metabolic syndrome [10].

With regards to MAFLD treatments, C-C chemokine receptor type 5 (CCR5) blockade and insulin sensitization are two postulated mechanisms, working on different inflammatory pathways. Maraviroc (MVC) is a licensed, host-directed drug for treating PWH with R5-tropic virus. Trials of MVC as an anti-inflammatory were conducted in rheumatoid arthritis [11], graft-versus-host-disease [12], and COVID-19 [13]. With regards to its role in NAFLD (now MAFLD), MVC protected against hepatic fat accumulation in a mouse model fed high-fat meals [14]. Similarly, the MAICOL study found a significant reversal of liver fibrosis when PWH and hepatitis C had their ritonavir-boosted protease inhibitor regimen intensified with MVC [15]. More recently, Cenicriviroc, a CCR2/CCR5 blocker, demonstrated antifibrotic effects without an impact on steatohepatitis [16], suggesting that the pathway of hepatic fat inflammation and hepatic fibrosis is different. However, a Phase III follow on trial [17] was halted early at the interim analysis, as it failed to show efficacy with regards to liver fibrosis in those with NASH (now renamed MASH).

The way in which metformin impacts on metabolic pathways is not fully understood. While we know metformin (MET) increases insulin-stimulated glucose uptake in the skeletal muscle and fatty acid oxidation in adipose tissue, and it is these pathways that are disrupted as part of the pathogenesis of MAFLD [18,19], there is evidence too that metformin may directly impact on the neurohormonal pathways that regulate appetite, and have a favorable effect on the microbiome [20]. In addition, MET is increasingly recognized to have anti-inflammatory properties via an effect on inhibition of nuclear factor κB via both the AMP-activated protein kinase dependent and independent pathways [21,22].

The aim of MAVMET, a randomized, open-label trial, with a 2 x 2 factorial design, was to determine whether adjunctive MVC, MET, or the combination of both over 48 weeks, compared to ART-alone was associated with significant reductions in liver fat fraction (LFF) in PWH, measured using MR PDFF.

## Materials and methods

### Study design and participants

MAVMET was an open-label, 48-week multicenter, randomized controlled factorial trial conducted across six sites in London, UK. Participants were allocated to one of four arms based on assignment of the two interventions: adjunctive MVC, MET or both in addition to ART, or ART-alone. MR PDFF images were gathered according to the MAVMET scanning protocol (Text, Supplemental Digital Content 2, radiology methodology) at baseline and week 48. All scans were performed on the same scanner at University College London Hospital (UCLH) and read by the same two blinded radiologists.

Note this trial was designed for NAFLD prior to the revised definitions for MAFLD which were introduced in 2023, otherwise, BMI in the overweight or obese range would have been inclusion criteria. Eligibility criteria were at least 35 years old, living with HIV for at least 5 years, on ART and virologically suppressed (<50 copies/ml) for at least 1 year, and with one feature suggestive of hepatic steatosis: more than 1 alanine transaminase (ALT) or aspartate aminotransferase (AST) above the upper limit of normal (ULN) without alternative explanation; increased waist circumference [≥94 cm (≥90 cm if South Asian origin) in men, ≥80 cm in women] (www.idf.org); or confirmed diagnosis of NAFLD on liver biopsy, imaging (computed tomography/MRI/ultrasound), or elastography. Exclusions: hepatitis B/C coinfection, other chronic liver disease, daily alcohol more than 20 g women and more than 30 g for men, B12 deficiency, current administration of MVC or MET, and ALT at least 10 ULN.

Ethical approval was authorized by the London-Hampstead Research Ethics Committee (17/LO/0998). MAVMET was granted Clinical Trial Authorisation (CTA# 00316/0247/001–0001) and was registered at ClinicalTrials.gov (NCT#: 03129113) and with EudraCt (2016–003575-21). All participants provided written informed consent prior to study procedures.

### Randomization

Eligible participants were randomized 1 : 1:1 : 1 to four arms: MVC (150, 300, or 600 mg twice daily) dose-adjusted for ART in accordance with the product information, MET 500 mg twice daily, MVC+MET (dosing as per the individual arms) or control (ART alone). Randomization was performed immediately at randomization visits, upon verification of eligibility from sites, using a centralized computer-generated system, independent of site investigators. Permuted blocks of varying size and sequences, stratified by recruiting center, were generated, encrypted, and stored centrally at the MRC CTU at UCL.

### Procedures

Participants eligible for enrolment at screening were randomized within 45 days at the baseline (week zero) visit [median length between screening and randomization 29 days (IQR 21–35)], with follow-up assessments at approximately weeks 4, 12, 24, 36, and 48. The week zero MR PDFF was to be performed between screening and the week zero visit [median length between scan and randomization 12 days (IQR 6–18)] due to the logistics of scanner availability and to allow safety review of images prior to commencing randomization. Follow-up MR PDFF was due at week 48 (±6 weeks). Weeks 0, 24, and 48 were the core visits which collected laboratory samples (hematology, biochemistry [including fasted lipids and insulin], HIV viral load, T-cell subsets, coagulation markers, plasma storage); 7-day adherence recall (based on the Terry Beirn Community Programs for Clinical Research on AIDS [CPCRA] Antiretroviral Medication Self-Report Form 065-BAS-2 and validated in the SMART trial; 23) (Image, Supplemental Digital Content 3, adherence questionnaire), alcohol intake (Image, Supplemental Digital Content 4, alcohol questionnaire), and EQ-5D quality-of-life questionnaires; weight; and waist circumference. A sleep questionnaire (modified Pittsburgh), Framingham cardiovascular risk score, and neck circumference were also assessed at week 0 and 48. Plasma was processed and stored at -70^o^C. Week 12 and 36 visits primarily focused on adverse event assessment.

### Radiology procedures

Complete MRI procedures are outlined in the supplemental (text, Supplemental Digital Content 2, radiology methodology). The analysis of scan data was performed separately to the unit where the scans were undertaken; all staff involved in performing scans or analyzing scan data were blinded to participant allocation. The constituents of the MRIs included MR PDFF; T1 measurement for liver fibrosis assessment; MRI slice at L4; whole body fat quantification; and ^1^H MRS. Hepatic steatosis grades using the LFF from the MRI were defined as grade 0: less than 6.4%, one: 6.4–17.3%, two: 17.4–22.1%, three: more than 22.1% [23].

### Mitigation strategy for the severe acute respiratory Syndrome Coronavirus-2 (SARS-CoV-2) pandemic

The emergence of COVID-19 in the first quarter of 2020 disrupted trial activities due to public health lockdowns. A protocol amendment and mitigation strategy was released in April 2020 (text, Supplemental Digital Content 22, MAVMET Protocol v2.0 22-April-2022, page 41). Some trial visits were conducted virtually by telephone which incurred some data loss including lab samples. Participants were offered reconsent, extension in the trial and continuation of study drug (if allocated) until their delayed week 48 scans. All participants were established on their arms for 20–48 weeks at the time of the amendment.

### Outcomes

The primary outcome was change in LFF between baseline and week 48 scans, as measured by MR PDFF. The secondary outcomes were review any change in the grade of hepatic steatosis by MR PDFF; assess impact on liver enzymes, HIV viral load, CD4^+^ and CD8^+^ T-cells; safety data, quality-of-life and medication adherence (7-day recall); and use of potentially hepatotoxic concomitant medications. Exploratory outcomes included changes in anthropomorphic assessments; sleep quantity/quality; cardiovascular risk (Framingham score); alcohol use; liver fibrosis scores and gradings; and extrahepatic fat measurements including pancreatic, total body, and visceral/subcutaneous adipose tissue using MR PDFF.

### Statistical analysis

A sample size of 90 participants (≈22 in each study arm) was estimated to give 80% power (at 5% significance level) based on the assumption that LFF would increase by at least 30% in the placebo group, decrease by 30–40% in the MVC and MET groups, and decrease by at least 40% in the MVC+MET group. These estimates were derived from observations of tesamorelin, a growth hormone analogue, on LFF when measured by ^1^H MRS [24].

The primary analysis was an analysis of covariance (ANCOVA), adjusting the 48-week value for baseline value, both randomized drugs as indicator variables, and a binary variable to indicate whether a participant had a delayed week 48 scan (i.e., beyond 54 weeks). The Hetregress command was used to allow for heteroskedasticity (i.e., increasing variance with higher baseline value). Pairwise interactions between the two study drugs and whether the scan was delayed were fitted, but are not included, in the final model due to lack of significance. Continuous secondary outcomes were analyzed using the same approach. Change in grade of hepatic steatosis was assessed by the Kruskal--Wallis test. Adverse events are reported as a percentage of participants experiencing them per treatment arm. All analyses were performed in (Stata v16.1: StataCorp LLC, College Station, TX, USA, 2020) [25].

## Results

**Fig. 1:**
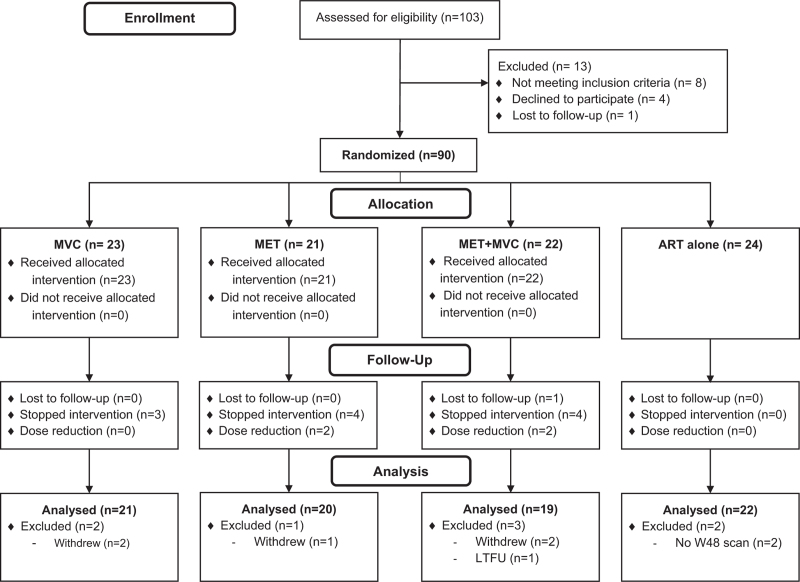
CONSORT statement.

Ninety participants were randomized (23 MVC, 21 MET, 22 MVC+MET, 24 ART alone) between March 19, 2018, and November 11, 2019 (Fig. 1). Five individuals withdrew (two MVC, one MET, two MVC+MET), two in the control arm undertook the week 48 visit but not the scan, and one MVC+MET participant was lost to follow-up. Eighty-two participants (21 MVC, 20 MET, 19 MVC+MET, 22 ART alone) had both a week 0 and week 48 (or delayed) scan and are included in the primary analysis. Of the week 48 scans, 20 (24%; seven MVC, four MET, four MVC+MET, five ART alone) were delayed beyond the protocol scheduled window (±6 weeks) due to the SARS-CoV-2 pandemic. The timing of the delayed scans ranged from 55 to 75 weeks (last scan was November 5, 2020), with a median additional period of 16 weeks.

Baseline characteristics are summarized in Table 1. Most participants (70%) were eligible for the trial based on imaging (mainly ultrasound) or biopsy-confirmed fatty liver disease, plus another criteria. Median age was 52 years (interquartile range, IQR 47–57), 93% male, 81% white ethnicity. Median CD4^+^ T-cell count, weight, BMI, and waist circumference were 672 cells/μl, 87 kg, 28 kg/m^2^, and 104 cm, respectively. Participants had a median baseline homeostatic model assessment for insulin resistance (HOMA-IR) of 2.9, with more than 2.9 being indicative of significant insulin resistance. Almost half (48%) of the cohort were on an integrase strand-transfer inhibitor (INSTI)-based regimen, 36% on nonnucleoside reverse transcriptase (NNRTI), and 17% on a ritonavir-boosted protease inhibitor (PI/r). Nineteen percent were taking tenofovir alafenamide (TAF) in their ART-backbone. There were high rates of current or prior hypertension (27%), hypercholesterolemia (43%), depression (46%), insomnia (30%), recreational drug use (37%), and cigarette smoking (56% current/past, 20% current). The median baseline Framingham score percentage was 5.5 (IQR 3.5–8.9).

**Table 1:** MAVMET baseline characteristics.

Characteristic	–	MVC	MET	MVC+MET	ART alone	Total
Total randomized	*n* (%)	23 (26)	21 (23)	22 (24)	24 (27)	90 (100)
Age (years)	Median (IQR)	50 (46–57)	52 (48–55)	54 (48–58)	52 (47–56)	52 (47–57)
Male	*n* (%)	22 (96)	21 (100)	20 (91)	21 (88)	84 (93)
Ethnicity	–	–	–	–	–	–
White	*n* (%)	20 (87)	16 (76)	20 (91)	17 (71)	73 (81)
Black African	*n* (%)	2 (9)	3 (14)	2 (9)	2 (8)	9 (10)
Hispanic/Latino	*n* (%)	1 (4)	1 (5)	0 (0)	1 (4)	3 (3)
Mixed ethnic group	*n* (%)	0 (0)	1 (5)	0 (0)	1 (4)	2 (2)
Black Caribbean/American	*n* (%)	0 (0)	0 (0)	0 (0)	1 (4)	1 (1)
Other	*n* (%)	0 (0)	0 (0)	0 (0)	2 (8)	2 (2)
Weight (kg)	Median (IQR)	87 (85–95)	91 (79–114)	88 (82–100)	84 (80–97)	87 (82–100)
BMI (kg/m^2^)	Median (IQR)	28 (27–31)	29 (26–37)	29 (27–33)	28 (26–30)	28 (26–33)
Waist circumference (cm)	Median (IQR)	103 (97–110)	107 (97–123)	105 (98–116)	103 (98–108)	104 (97–112)
Neck circumference (cm)	Median (IQR)	41 (39–44)	43 (39–44)	43 (40–44)	42 (39–44)	42 (39–44)
Framingham score (%)	Median (IQR)	5.7 (4.0–7.9)	5.1 (4.3–7.3)	6.4 (3.4–10.2)	5.0 (2.5–8.0)	5.5 (3.5–8.9)
CD4^+^ T-cell count (cells/μl) *n* = 89	Median (IQR)	680 (550–880)	650 (390–730)	673 (515–890)	659 (470–831)	672 (451–831)
Time since HIV diagnosis (years)	Median (IQR)	13 (8–17)	17 (10–20)	14 (9–20)	15 (11–20)	15 (9–20)
Time since ART initiation (years)	Median (IQR)	9 (6–16)	10 (6–17)	11 (8–17)	13 (7–18)	11 (6–17)
Baseline ART	–	–	–	–	–	–
INSTI^a^	*n* (%)	10 (43)	7 (33)	16 (73)	10 (42)	43 (48)
NNRTI	*n* (%)	8 (35)	12 (57)	6 (27)	6 (25)	32 (36)
PI	*n* (%)	5 (22)	2 (10)	0 (0)	8 (33)	15 (17)
Taking TAF	*n* (%)	3 (13)	3 (14)	7 (32)	4 (17)	17 (19)
Entry criteria	–	–	–	–	–	–
Scan or biopsy diagnosis plus ≥1 other criteria	*n* (%)	17 (74)	14 (67)	13 (59)	19 (79)	63 (70)
Scan or biopsy diagnosis only	*n* (%)	0 (0)	2 (10)	0 (0)	0 (0)	2 (2)
Raised ALT/AST + waist circumference	*n* (%)	0 (0)	1 (5)	3 (14)	0 (0)	4 (4)
Raised waist circumference only	*n* (%)	6 (26)	4 (19)	5 (23)	5 (21)	20 (22)
Raised ALT/AST only	*n* (%)	0 (0)	0 (0)	1 (5)	0 (0)	1 (1)
Medical history (reported by participant, verified in medical record)	–	–	–	–	–	–
Depression	*n* (%)	6 (26)	7 (33)	14 (64)	14 (58)	41 (46)
Hypercholesterolemia	*n* (%)	7 (30)	10 (48)	13 (59)	9 (38)	39 (43)
Insomnia	*n* (%)	6 (26)	6 (29)	7 (32)	8 (33)	27 (30)
Hypertension	*n* (%)	8 (35)	4 (19)	6 (27)	6 (25)	24 (27)
Obstructive sleep apnea	*n* (%)	0 (0)	1 (5)	2 (9)	0 (0)	3 (3)
Diabetes	*n* (%)	0 (0)	1 (5)	0 (0)	0 (0)	1 (1)
Alcoholism/alcohol abuse	*n* (%)	1 (4)	2 (10)	1 (5)	1 (4)	5 (6)
Illicit substance abuse	*n* (%)	8 (35)	11 (52)	8 (36)	6 (25)	33 (37)
Current smoker	*n* (%)	4 (17)	3 (14)	4 (18)	7 (29)	18 (20)
Past smoker	*n* (%)	8 (35)	11 (52)	4 (18)	9 (38)	32 (36)
Baseline laboratory results	–	–	–	–	–	–
ALT (U/l, *n* = 89)	Median (IQR)	41 (35–56)	39 (30–69)	43 (35–65)	38 (27–52)	41 (31–61)
Total cholesterol (mmol/l)	Median (IQR)	5.0 (4.0–5.9)	4.7 (4.4–5.1)	4.6 (4.2–5.8)	4.5 (3.7–5.1)	4.6 (4.1–5.5)
HOMA-IR (*n* = 83)	Median (IQR)	3.2 (1.7–5.7)	2.7 (1.7–6.0)	3.4 (1.8–5.4)	2.7 (2.0–4.1)	2.9 (1.8–5.5)
MRI	–	–	–	–	–	–
Liver fat fraction (%)	Mean (SD)	8.7 (6.3)	13.5 (9.5)	13.2 (9.1)	11.8 (9.4)	11.8 (8.7)
Hepatic steatosis grade (% liver fat)	–	–	–	–	–	–
0 (0–6.3)	*n* (%)	11 (48)	7 (33)	8 (36)	10 (42)	36 (40)
1 (6.4–17.3)	*n* (%)	11 (48)	6 (29)	8 (36)	9 (38)	34 (38)
2 (17.4–22.0)	*n* (%)	0 (0)	5 (24)	2 (9)	0 (0)	7 (8)
3 (≥22.1)	*n* (%)	1 (4)	3 (14)	4 (18)	5 (21)	13 (14)

ALT, alanine transaminase; ART, antiretroviral therapy; AST, aspartate transaminase; HOMA-IR, homeostatic model assessment for insulin resistance; INSTI, integrase strand transfer inhibitor; MET, metformin; MVC, maraviroc; NNRTI, nonnucleoside reverse transcriptase inhibitor; PI, protease inhibitor; SD, standard deviation; TAF, tenofovir alafenamide fumarate.

a Of those on INSTI (*n* = 43), 24 (56%) were on raltegravir, 15 (35%) on dolutegravir, and 4 (9%) on elvitegravir.

**Fig. 2:**
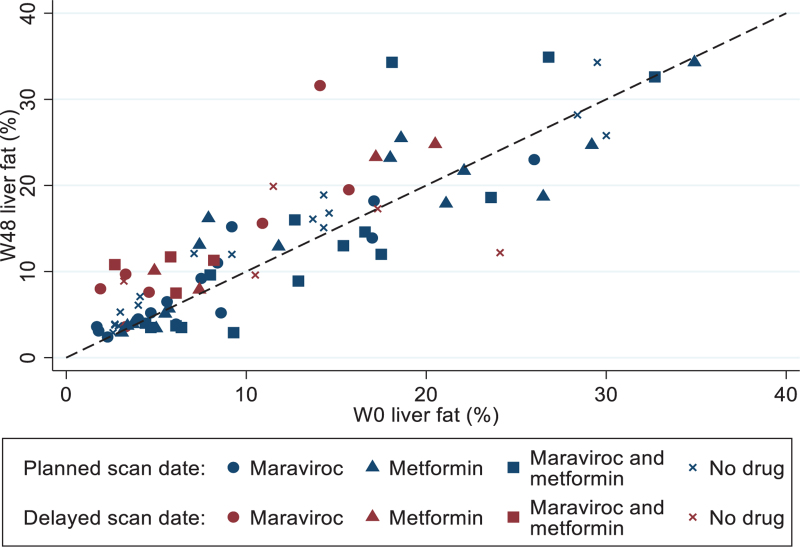
Scatter plot of W0 liver fat versus W48 liver fat measured using magnetic resonance proton density fat fraction.

Mean baseline LFF was 11.8% (SD 8.6). However, the distribution of baseline LFF values was markedly right-skewed (Fig. 2): median baseline LFF was 8.9% (IQR 4.6–17.1) with a chance imbalance by treatment arm (7.5% MVC, 9.8% MET, 11.0% MVC+MET, 9.8% control arm; there was no imbalance in mean baseline LFF). There was a small increase in mean LFF between the baseline and follow-up MRI scans in all arms: 2.22% MVC, 1.26% MET, 0.81% MVC+MET, and 1.39% control arm (Table 2). In analysis of covariance, there was no significant difference in the predicted change for any of the intervention arms compared to ART alone (MVC -0.42% [95% CI -1.53, +0.68, *P* = 0.45], MET -0.62% [-1.81, +0.56, *P* = 0.30], MVC+MET -1.04% [-2.74, + 0.65, *P* = 0.23]; Table 2).

The black dashed line is a y = x reference line. Points below this line represent participants who reduced their liver fat; points above this line represent participants who increased their liver fat.

**Table 2:** Primary analysis of the change in liver fat measured using magnetic resonance proton density fat fraction.

Study arm	Sample	Mean (SD) change in LFF (%) at 48 weeks	Absolute change LFF (%)	*P*
ART alone	22	1.4 (4.0)	0 [Reference]	–
MVC	21	2.2 (4.6)	−0·42 (−1.53, 0.68)	*P* = 0.454
MET	20	1.3 (4.1)	−0.62 (−1.81, 0.56)	*P* = 0.304
MVC + MET	19	0.8 (5.7)	−1.04 (−2.74, 0.65)	*P* = 0.227

ART, antiretroviral therapy; LFF, Liver fat fraction; MET, Metformin; MVC, maraviroc; SD, standard deviation.

Participants with a delayed week 48 MRI scan showed a much larger increase in LFF compared with those who were scanned as scheduled in the protocol (estimated difference 4.23% [95% CI 2.97–5.48], *P* < 0.001). An analysis limited to participants whose scans were timed as per protocol found increases in mean LFF between the baseline and week 48 scans of 0.35% for MVC, 0.56% for MET, 1.72% for the control arm, and a decrease of 0.21% for MVC+MET. Predicted effects of change in LFF from analysis of covariance were -0.69% (95% CI -1.64, 0.25, *P* = 0.15) for MVC, -1.45% (-2.52, -0.38, *P* = 0.008) for MET, and -2.14% (-3.66, -0.63, *P* = 0.005) for MVC+MET (Table 2, Figure 1S, Supplemental Digital Content 13, scatter plot). A second sensitivity analysis, limited to participants with baseline grade 1–3 steatosis (but including delayed scans), showed broadly similar findings to the primary analysis (Table 2), that is, no evidence of an effect of either investigational drug. Mean weight change was -0.46, -0.63, + 0.48 kg in those with standard duration of follow-up prepandemic, standard duration of follow-up during the pandemic, and those with extended follow-up, respectively, but with the caveat that the numbers (see above) in each arm become small.

Most participants had grade zero (40%) or grade one (38%) hepatic steatosis at baseline (Table 1). For those scanned at both timepoints (*n* = 81), 56 (68%) had no change in hepatic steatosis grading, while 18 (22%) increased by one grade and two (2%) increased by two grades (Table 3). There was no evidence of difference between study arms (*P* = 0.55). There were no significant differences in the change in liver enzymes (ALT, AST, gamma-glutamyl transpeptidase) in the intervention arms compared to the control (Figure 2S-4S, Supplemental Digital Content 14–16, plots of liver enzymes over time). There was a mild decrease in alkaline phosphatase (ALP) for MVC+MET compared to ART alone (-13 U/l [-21,-5], *P* = 0.002, Table 8S and Figure 5S, Supplemental Digital Content 13 and 17, change in ALP and plot of ALP over time). Use of hepatotoxic medications was modest (*n* = 39/90, 43%), mostly atorvastatin (24/39, 62%). There were four nonsustained viral load blips (>50 copies/ml), three occurring at week 4, one at week 24, with no difference between arms (*P* = 0.84).

**Table 3:** Change in grade of hepatic steatosis as measured by MR PDFF.

	MVC	MET	MVC + MET	ART alone	Total
Sample	21 (26)	20 (24)	19 (23)	22 (27)	82 (100)
Change in grade	–	–	–	–	–
−2	0 (0)	0 (0)	0 (0)	1 (5)	1 (1)
−1	1 (5)	1 (5)	3 (16)	0 (0)	5 (6)
No change	13 (62)	14 (70)	12 (63)	17 (77)	56 (68)
+1	6 (29)	4 (20)	4 (21)	4 (18)	18 (22)
+2	1 (5)	1 (5)	0 (0)	0 (0)	2 (2)

	*P*
Four arm comparison	*P* = 0.547
Just maraviroc arms	*P* = 0.865
Just metformin arms	*P* = 0.556

All values are *n* (%). ART, antiretroviral therapy; MET, metformin; MR PDFF, magnetic resonance proton density fat fraction; MVC, maraviroc.

Participant reported adherence, a 7-day recall (Image, Supplemental Digital Content 3, Adherence questionnaire) captured at every visit, was reasonably high, with the majority taking all their pills or most of their pills over the prior 7 days, and no difference between arms (Table 1S, Supplemental Digital Content 5, Adherence summary). The additional pill burden in the intervention arms did not negatively impact adherence to ART. In a per-protocol analysis of those taking more than 50% (Table 2S, Supplemental Digital Content 6, per-protocol analysis) or more than 75% (Table 3S, Supplemental Digital Content 7, per-protocol analysis) of their treatment, there was no significant difference in absolute or relative change of LFF.

Six serious adverse events (SAEs) occurred. Three in the MVC+MET arm: prostate cancer and influenza pneumonia both deemed unrelated to both drugs, and one biopsy-confirmed pancreatic adenocarcinoma (this participant subsequently died after completing the trial) considered by the Investigator to be unrelated to MET and unlikely related to MVC. The other Grade three and four AEs are described in Table 4S, Supplemental Digital Content 8, Adverse Events. 23 episodes of treatment-limiting toxicity occurred in 11 participants in the intervention arms (two MVC, five MET, four MVC+MET). Diarrhea/loose stools was the most common complaint (*n* = 11/23, 48%), with a further seven events (30%) also being gastrointestinal-associated side effects. Treatment was stopped for five participants, with the remainder undergoing treatment interruptions.

CD4^+^ and CD8^+^ T-cell counts were stable apart from the MVC+MET arm where there was a significant increase in median absolute CD4^+^ T-cells of 89 cells/μl (IQR 12–166, *P* = 0.024, Table 5S Supplemental Digital Content 9, change in CD4^+^ table, and Figure 6S, Supplemental Digital Content 18, plot of CD4^+^ over time) and CD8^+^ T-cells of 128 cells/μl (20–236, *P* = 0.021, Table 6S, Supplemental Digital Content 10, change in CD8^+^ table, and Figure 7S, Supplemental Digital Content 19, plot of CD8^+^ over time), without any significant change in CD4^+^ and CD8^+^ T-cell percentages. There were no discernible differences in median self-reported total health score (0, IQR -10,+7, *P* = 0.627) or any of the quality-of-life subsections of the EQ-5D questionnaire.

In the exploratory analyses, there were no differences in lipid markers, insulin resistance, waist and neck circumference, or Framingham score. There was a trend toward weight loss in the MET arm by week 24 (mean reduction 2.0 kg [-4,0], *P* = 0.08); this was not maintained by week 48 (mean reduction -1 kg [-3,1], *P* = 0.29, Table 7S, Supplemental Digital Content 11, table of weight change by arm, and Figure 8S, Supplemental Digital Content 20, weight change by arm over time).

## Discussion

Baseline LFF as measured on MRI was lower than expected in this cohort of older PWH, and the majority had only grade zero or one hepatic steatosis. However, median baseline HOMA-IR was at the threshold of insulin resistance; 60% had abdominal obesity (waist circumference >102 cm) and 90% were in the overweight or obese BMI range at baseline, demonstrating the participants did have several features of the metabolic syndrome. Overall, there was no significant change in liver fat percentage as measured by MR PDFF when taking ART with adjunctive maraviroc, metformin, or the combination of both, compared to ART alone.

Paradoxically, longer exposure to study drugs was associated with increased liver fat, not fully explained by other factors including weight gain during COVID-19 lockdowns, however numbers are quite small, and should be interpreted with caution. The interventions were reasonably well tolerated, although gastrointestinal upset was a rate-limiting toxicity in the MET and MVC+MET arms, driven by the MET which was administered as the standard formulation. We were cautious about the MET dose because of interaction concerns with dolutegravir when designing the trial. The slow-release formulation would likely have better tolerability even at higher doses, and it is possible that higher doses may yield a reduction in liver fat.

CD4^+^ and CD8^+^ T-cells increased only in the MVC+MET arm, an unexpected finding warranting further exploration. Increases in these T-cell subsets have been reported with maraviroc and represent a short-lived (usually during the first 3–6 months of exposure) trafficking phenomenon [26]. However, this does not seem to be the explanation here, as the increases continued from week 24 to week 48, and if maraviroc were the key driver of this, this would have been replicated in the maraviroc-only arm.

Key limitations of this study include, the largely homogenous cohort (males of white ethnicity) limiting the generalizability. Second, although very sensitive, MR PDFF becomes less reliable in discerning changes in LFF below 5% and is unable to distinguish steatosis from steatohepatitis. Similarly, we note the surrogate markers of fatty liver disease used to enroll participants in the trial were not very sensitive, even though 70% of individuals had radiological or histological evidence of fatty liver disease. We deliberately avoided screening with MRI due to cost. Mandated screening using Fibroscan may have increased sensitivity for detecting significant steatosis. Finally, when calculating the sample size, we hypothesized a greater increase in LFF in the control arm than was actually observed. This implies that the trial was statistically under-powered, although the interpretation of the confidence intervals around the treatment effects remains valid [27].

Regarding future directions, there is promising data on semaglutide, a glucagon-like peptide-1 receptor agonist, which has displayed benefits in terms of weight and liver fat reductions [28,29]. This seems an obvious next step for PLWH. Challenges in MAFLD research continues to include the improvement of radiological techniques and biomarkers to identify individuals with MAFLD or MASH. Similarly, patients with MASH are a key subpopulation to differentiate. Due to the increased risk of irreversible cirrhosis, this group could potentially benefit the most from antiinflammatories or drug combinations. Drug combinations would ideally include agents with weight loss effects. Medical management should be supplemented with robust nonpharmacological weight loss programs to address lifestyle factors that could be influencing the disease processes underpinning MAFLD and MASH.

## Acknowledgements

The MAVMET Study Group:

MAVMET trial team at MRC CTU at UCL: Helen Webb, Claire Murphy, Leanne McCabe, Anna Goodman, Adam Gregory, Yolanda Collaco-Moraes, Mary Rauchenberger, Fatima Mohamed, Aminata Sy, David Dunn, Anna Turkova, Hannah Vaughan, Chiara Borg.

Clinical sites:

Mortimer Market Centre: Gaynor Lawrenson, Marzia Fiorino, James Burns, Erica Pool, Pierre Pellegrino, Alejandro Arenas-Pinto, Maria Muller Nunez, Hinal Lukha, Richard Gilson.

Guys & St Thomas’ Hospital: Julie Fox, Hiromi Uzu, Julianne Lwanga, Ming Lee, Fiona Ryan, Venkateshwaran Sivaraj, Andrea Berlanga, Jessica Doctor.

Royal Free Hospital: Jonathan Edwards, Margaret Johnson, Nnenna Ngwu, Tristan Barber, Alice Nightingale, Mike Youle, Sara Madge, Sabine Kinloch de Loes, Tom Fernandez.

St Mary's Hospital: Ian McGuinness, Lucy Garvey, Claire Petersen, Rebecca Hall, Wilbert Ayap, Jasmini Alagaratnam.

King's College Hospital: Frank Post, Beatriz Santana, Lucy Campbell, Ana Canoso, Kate Childs, Maria Liskova, Verity Sullivan, Kate Flanagan, John Phelan, Chris Taylor.

Royal London Hospital: Chloe Orkin, Anele Waters, Chris Clarke, James Hand, John Thornhill, Simon Rackstraw, Rebecca Marcus.

University College London Hospital Radiology: Manil Chouhan, Arash Latifoltojar, Shonit Punwani.

ViiV Healthcare: Rekha Trehan, Andrew Clark.

Royal Free Hospital Central Pharmacy: Sabina Melander.

Independent Clinical Reviewers: Brian Angus, Christoph Boesecke.

Trial Steering Committee: Graham Cooke, Jason Baker, Steve Ryder.

Trial Steering Committee Community Representative: Chris Sandford.

The authors thank all participants for their contribution to MAVMET.

They acknowledge and thank our dear colleague Izabela Tolowinska (who passed away in 2023) for her enduring support.

Lm.C. and D.D. did the statistical analysis. J.E.B. and S.L.P. wrote the first draft of the report with input from D.D., J.B., Lm.C., F.P., L.G., J.F., S.R., B.A., C.B., C.O., A.G., A.C. B.A. and C.B. were in addition clinical reviewers for the adverse events. A.L., M.C., and S.P. reviewed the manuscript an ensured details of radiological methodology were correct. S.R., J.B., G.C. led the Trial Management Group and inputted into the final report and this manuscript. All other listed co-authors delivered the trial at the Clinical trials unit (S.L.P., D.D., Lm.C., A.G., Y.C.-M., M.R., C.M., H.W., A.G., F.M.) or the sites (S.L.P., J.E.B., F.P., J.F., E.P., A.W., B.S., L.G., M.J., I.Mc.G., J.E., C.O.). C.S. (deceased) was our community representative and an active member of the Trial Management Group. All authors had full access to all the data in the study (presented at a results meeting) and had final responsibility for the decision to submit for publication.

These data were presented at the 18^th^ European AIDS Conference, London, UK (27–31 October 2021) and received a best poster presentation award.

Data will be shared according to the ICTM's controlled access approach, based on the following principles:

No data should be released that would compromise an ongoing trial or study.

There must be a strong scientific or other legitimate rationale for the data to be used for the requested purpose.

Investigators who have invested time and effort into developing a trial or study should have a period of exclusivity in which to pursue their aims with the data before key trial data are made available to other researchers.

The resources required to process requests should not be under-estimated, particularly successful requests, which lead to preparing data for release. Therefore, adequate resources must be available in order to comply in a timely manner or at all, and the scientific aims of the study must justify the use of such resources.

Data exchange complies with Information Governance and Data Security Policies in all the relevant countries.

Data will be available for sharing from 2023 onwards. Researchers wishing to access MAVMET data should contact the Trial Management Group in the first instance.

This work was supported by ViiV Healthcare, UK, via an independent academic grant (ViiV Reference Etrack Number 204817). Salary support is provided from the Medical Research Council, United Kingdom (grant MC_UU_00004/03 to S.L.P. and MC_UU_00004/04 to D.D.).

The supply of maraviroc for the study was provided by ViiV Healthcare and the metformin was supplied by the UK National Health Service, labelled and QP released by the Royal Free Hospital Clinical Trials Pharmacy. ViiV Healthcare had no role in the collection, analysis, or interpretation of the trial data; they reviewed the final manuscript prior to submission.

### Conflicts of interest

There are no conflicts of interest.

## Supplementary Material

Supplemental Digital Content
