# Supplementary material for: MAVMET trial: maraviroc and/or metformin for metabolic dysfunction associated fatty liver disease in adults with suppressed HIV
Source: AIDS. 2024 Jun 25;38(10):1513–22. doi: 10.1097/QAD.0000000000003947 (PMC11239089; doi:10.1097/QAD.0000000000003947)
Supplement: Supplemental Digital Content [file aids-38-1513-s001.docx]

**SUPPLEMENTAL DIGITAL CONTENT**

**SDC1: MAVMET study group 2**

**SDC2: MAVMET radiology scanning protocol 3**

**Questionnaires**

**SDC3: Adherence questionnaire (7-day recall): completed at weeks 0, 4, 12, 24, 36 and 48. 4**

**SDC4: Alcohol questionnaire: completed at screening, weeks 0, 12, 24, 36 and 48. 5**

**Supplementary Tables**

**SDC5: Table 1S. Adherence summary and missed doses. 6**

**SDC6: Table 2S.** **Per-protocol analysis: those who took >50% of treatment 7**

**SDC7: Table 3S. Per-protocol analysis: those who took >75% of treatment 7**

**SDC8: Table 4S. SAEs, Grade 3/4 AEs and treatment limiting toxicities 8**

**SDC9: Table 5S. Change in CD4+ T-cell count 9**

**SDC10: Table 6S. Change in CD8+ T-cell count 9**

**SDC11: Table 7S. Weight change by arm 9**

**SDC12: Table 8S. Change in ALP 10**

**Supplementary Figures**

**SDC13: Figure 1S**. **Scatter plot of W0 liver fat vs W48 liver fat: Planned scan date participants only 11**

**SDC14: Figure 2S. Plot of ALT over time 12**

**SDC15: Figure 3S. Plot of AST over time 12**

**SDC16: Figure 4S. Plot of GGT over time 13**

**SDC17: Figure 5S. Plot of ALP over time 13**

**SDC18: Figure 6S.** **Plot of CD4+ T-cells over time 14**

**SDC19: Figure 7S.** **Plot of CD8+ T-cells over time 14**

**SDC20: Figure 8S.** **Weight change by arm 15**

**SDC21: Radiology Protocol References 16**

**SDC22: MAVMET protocol (version 2.0 22-Apr-2020) 18**

**SDC1: MAVMET STUDY GROUP**

**MAVMET trial team at MRC CTU at UCL**: Helen Webb, Claire Murphy, Leanne McCabe, Anna Goodman, Adam Gregory, Yolanda Collaco-Moraes, Mary Rauchenberger, Fatima Mohamed, Aminata Sy, David Dunn, Anna Turkova, Hannah Vaughan, Chiara Borg

**Clinical sites:**

**Mortimer Market Centre:** Gaynor Lawrenson, Marzia Fiorino, James Burns, Erica Pool, Pierre Pellegrino, Alejandro Arenas-Pinto, Maria Muller Nunez, Hinal Lukha, Richard Gilson

**Guys & St Thomas’ Hospital:** Julie Fox, Hiromi Uzu, Julianne Lwanga, Ming Lee, Fiona Ryan, Venkateshwaran Sivaraj, Andrea Berlanga, Jessica Doctor

**Royal Free Hospital:** Jonathan Edwards, Margaret Johnson, Nnenna Ngwu, Tristan Barber, Alice Nightingale, Mike Youle, Sara Madge, Sabine Kinloch de Loes, Tom Fernandez

**St Mary’s Hospital**: Ian McGuinness, Lucy Garvey, Claire Petersen, Rebecca Hall, Wilbert Ayap, Jasmini Alagaratnam

**King’s College Hospital**: Frank Post, Beatriz Santana, Lucy Campbell, Ana Canoso, Kate Childs, Maria Liskova, Verity Sullivan, Kate Flanagan, John Phelan, Chris Taylor

**Royal London Hospital:** Chloe Orkin, Anele Waters, Chris Clarke, James Hand, John Thornhill, Simon Rackstraw, Rebecca Marcus

**University College London Hospital Radiology**: Manil Chouhan, Arash Latifoltojar, Shonit Punwani

**ViiV Healthcare**: Rekha Trehan, Andrew Clark

**Royal Free Hospital Central Pharmacy**: Sabina Melander

**Independent Clinical Reviewers**: Brian Angus, Christoph Boesecke

**Trial Steering Committee:** Graham Cooke, Jason Baker, Steve Ryder

**Trial Steering Committee Community Representative**: Chris Sandford†

†Our colleague and friend, Chris Sandford very sadly passed away in November 2022.

**SDC2: MAVMET RADIOLOGY SCANNING PROTOCOL**

Data and methodology used in developing the MRI-scanning protocol in MAVMET are detailed in References 1-14 inclusive (see SDC16).

**Image acquisition**

Following local MRI safety protocols, consented and enrolled patients were scanned on a single 3.0 Tesla wide-bore MR scanner (Ingenia; Phillips Healthcare, Best, the Netherlands). Full body coverage (vertex to feet) was obtained through a multi-station acquisition of contiguous body regions with the manufacturers' head coil, two anterior surface coils and table-embedded posterior coils.

As part of a whole-body MRI protocol, proton density fat fraction (PDFF) sequences (Philips mDixonQuant) were used to obtain images of the liver in all patients during a breath hold acquisition [3D spoiled gradient echo, TE: shortest (~1-2ms), TR: shortest (~5-10ms), slice thickness: 5mm, number of echoes: 6, flip angle: 3, acceleration factor (SENSE): 2, field of view: 510 x 300mm, slice thickness: 5mm, number of slices: 40, acquisition time: 17s] and fat-fraction maps were reconstructed from acquired data using vendor-based software.

**Image analysis**

Data was analysed by an abdominal radiologist (MC) and a radiology fellow (AL), both with >10 years experience in abdominal MRI imaging.

Initially, whole-body MRI images were reviewed by a clinical radiologist (MC) for unexpected incidental or adverse findings. Actionable findings were escalated to the clinical team and recorded as per the study protocol (supplementary section, MAVMET Protocol).

Anonymised fat-fraction maps of the upper abdomen were analysed using in-house developed MATLAB code. Single-slice, circular regions of interest (ROIs, up to 20mm in diameter) were placed on each of the liver segments (segments I-VIII) and fat-fraction values were derived for each segment. The mean of these was used for whole liver fat fraction. A non-alcoholic steatohepatitis (NASH) score was also assigned for each patient (grade 0 (fat fraction <6.4%), grade 1 (fat fraction 6.4-17.3%), grade 2 (fat fraction: 17.4-22.1%) and grade 3 (fat fraction >22.1%), based on whole liver fat fraction (1).

**QUESTIONNAIRES**

**SDC3: Adherence questionnaire (7-day recall): completed at weeks 0, 4, 12, 24, 36 and 48.**


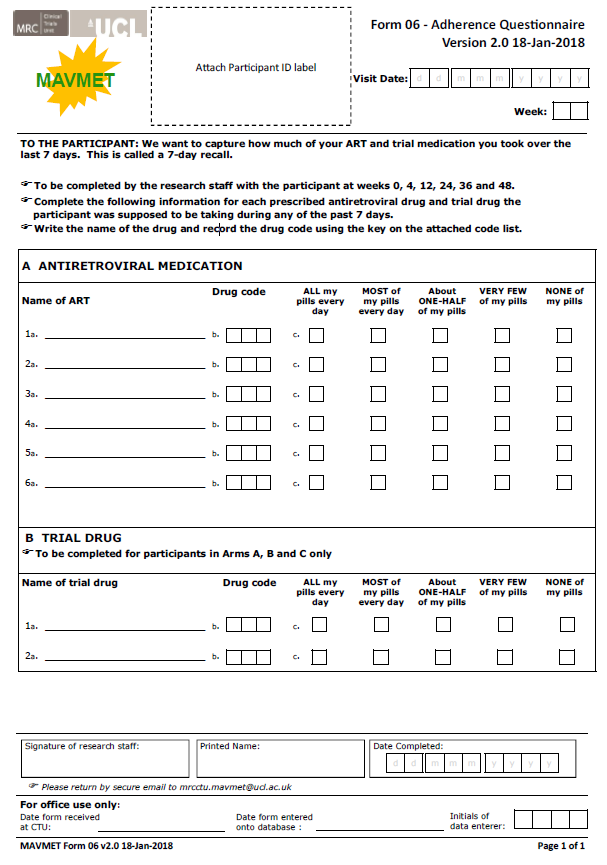


**SDC4: Alcohol questionnaire: completed at screening, weeks 0, 12, 24, 36 and 48.**


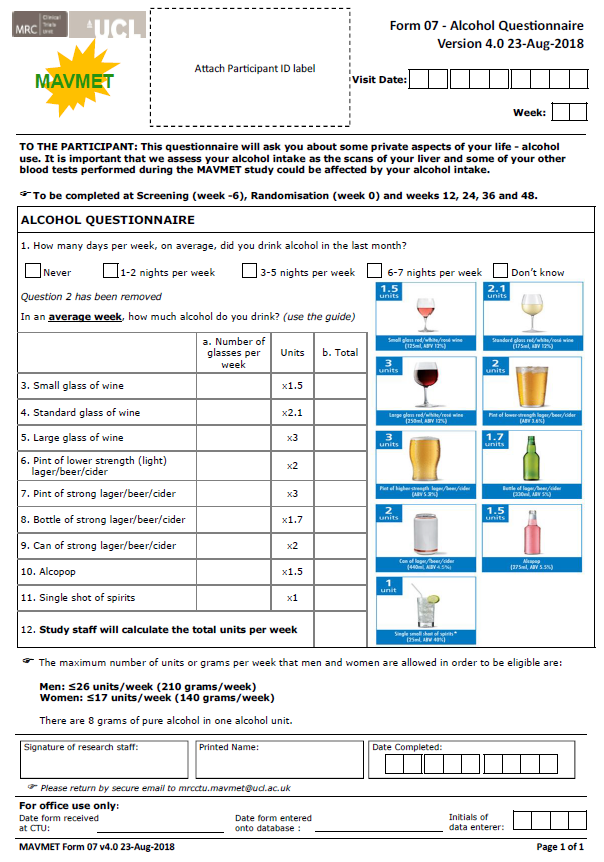


**SUPPLEMENTARY TABLES**

**SDC5: Table 1S. Adherence summary and missed doses.**

|  | Maraviroc | Metformin | Maraviroc and metformin | Total | p-value |
| --- | --- | --- | --- | --- | --- |
| Total randomised | N=23 | N=21 | N=22 | N=66 |  |
|  |  |  |  |  |  |
| Stopped treatment early | 3 (13%) | 4 (19%) | 4 (18%) | 11 (17%) | p=0.844 |
| Had ≥1 dose decrease [total number] | 0 [0] | 2 (10%) [2] | 1 (5%) [2] | 3 (5%) [3] | p=0.265 |
|  |  |  |  |  |  |
| Missed any doses | 10 (45%) | 6 (29%) | 10 (53%) | 26 (42%) | p=0.280 |
| Median (IQR) [range] % doses missed | 0 (0, 10) [0, 40] | 1 (0, 2) [0, 33] | 0 (0, 4) [0, 29] | 0 (0, 5) [0, 40] |  |
|  |  |  |  |  |  |
| Adherence at W4 | N=17 | N=20 | N=16 | N=53 |  |
| Took all pills | 13 (76%) | 18 (90%) | 14 (88%) | 45 (85%) | p=0.363 |
| Took most pills | 4 (24%) | 1 (5%) | 2 (13%) | 7 (13%) |  |
| Took half the pills | 0 | 1 (5%) | 0 | 1 (2%) |  |
|  |  |  |  |  |  |
| Adherence at W12 | N=19 | N=18 | N=17 | N=54 |  |
| Took all pills | 14 (74%) | 17 (94%) | 13 (76%) | 44 (81%) | p=0.229 |
| Took most pills | 5 (26%) | 1 (6%) | 4 (24%) | 10 (19%) |  |
|  |  |  |  |  |  |
| Adherence at W24 | N=20 | N=15 | N=17 | N=52 |  |
| Took all pills | 17 (85%) | 13 (87%) | 16 (94%) | 46 (88%) | p=0.943 |
| Took most pills | 2 (10%) | 2 (13%) | 1 (6%) | 5 (10%) |  |
| Took half the pills | 1 (5%) | 0 | 0 | 1 (2%) |  |
|  |  |  |  |  |  |
| Adherence at W36 | N=20 | N=16 | N=18 | N=54 |  |
| Took all pills | 16 (80%) | 13 (81%) | 15 (83%) | 44 (81%) | p=0.802 |
| Took most pills | 4 (20%) | 2 (13%) | 2 (11%) | 8 (15%) |  |
| Took no pills | 0 | 1 (6%) | 1 (6%) | 2 (4%) |  |
|  |  |  |  |  |  |
| Adherence at W48 | N=21 | N=17 | N=17 | N=55 |  |
| Took all pills | 16 (76%) | 15 (88%) | 11 (65%) | 42 (76%) | p=0.550 |
| Took most pills | 4 (19%) | 2 (12%) | 5 (29%) | 11 (20%) |  |
| Took no pills | 1 (5%) | 0 | 1 (6%) | 2 (4%) |  |

**SDC6: Table 2S****.** **Per-protocol analysis: those who took >50% of treatment.**

|  | Maraviroc | Metformin | Maraviroc and metformin | No drug (reference category) | Total |
| --- | --- | --- | --- | --- | --- |
| Total randomised | 22 (26) | 18 (22) | 19 (23) | 24 (29) | 83 (100) |
| Total with a W48 scan | 21 (26) | 18 (23) | 18 (23) | 22 (28) | 79 (100) |
| Mean (SD) liver fat at 48 weeks (%) | 10.5 (7.7) | 14.9 (9.9) | 13.9 (10.2) | 12.9 (8.7) | 12.9 (9.1) |
| Mean (SD) change in liver fat to 48 weeks (%) | 2.2 (4.6) | 1.8 (3.7) | 0.9 (5.8) | 1.4 (4.0) | 1.6 (4.5) |
| Absolute change (%) | -0.40 (-1.54, 0.74) p=0.490 | -0.59 (-1.82, 0.65) p=0.353 | -0.99 (-2.77, 0.80) p=0.278 | - | - |

Note: both models adjust for if the scan was performed as planned or delayed, p<0.001 in both models.

**SDC7: Table 3S. Per-protocol analysis: those who took >75% of treatment.**

|  | Maraviroc | Metformin | Maraviroc and metformin | No drug (reference category) | Total |
| --- | --- | --- | --- | --- | --- |
| Total randomised | 20 (26) | 16 (20) | 18 (23) | 24 (31) | 78 (100) |
| Total with a W48 scan | 19 (26) | 16 (21) | 17 (23) | 22 (30) | 74 (100) |
| Mean (SD) liver fat at 48 weeks (%) | 10.5 (7.7) | 13.1 (8.8) | 13.9 (10.6) | 12.9 (8.7) | 12.6 (8.9) |
| Mean (SD) change in liver fat to 48 weeks (%) | 2.4 (4.8) | 1.6 (3.8) | 1.1 (5.9) | 1.4 (4.0) | 1.6 (4.6) |
| Absolute change (%) | -0.15 (-1.07, 0.77) p=0.748 | -0.72 (-1.78, 0.33) p=0.180 | -0.87 (-2.38, 0.63) p=0.256 | - | - |

Note: both models adjust for if the scan was performed as planned or delayed, p<0.001 in both models.

## **SDC8: Table 4S. SAEs, Grade 3/4 AEs and treatment limiting toxicity.**

| - | **MVC** | **MET** | **MVC + MET** | **ART Alone** | **Total** | **p-value** |
| --- | --- | --- | --- | --- | --- | --- |
| Sample | 23 (22) | 29 (28) | 27 (26) | 24 (24) | 103 (100) | - |
| - | - | - | - | - | - | - |
| SAEs | 2 (9) | 1 (5) | 3 (14) | 0 (0) | 6 (7) | p=0.269 |
| Life-threatening | 0 | 0 | 3 (100) | - | 3 (50) | - |
| Required hospitalisation | 1 (50) | 1 (100) | 0 (0) | - | 2 (33) | - |
| Other important medical condition | 1 (50) | 0 (0) | 0 (0) | - | 1 (17) | - |
| Relation to maraviroc | - | - | - | - | - | - |
| Unlikely | 0 (0) | - | 1 (33) | - | 1 (20) | - |
| Not related | 2 (100) | - | 2 (67) | - | 4 (80) | - |
| Relation to metformin | - | - | - | - | - | - |
| Not related | - | 1 (100) | 3 (100) | - | 4 (100) | - |
| - | - | - | - | - | - | - |
| Grade 3 or 4 AEs | 1 (4) | 1 (5) | 3 (14) | 1 (4) | 6 (7) | p=0.650 |
| Relation to maraviroc | - | - | - | - | - | - |
| Unlikely | 0 (0) | - | 1 (33) | - | 1 (25) | - |
| Not related | 1 (100) | - | 2 (67) | - | 3 (75) | - |
| Relation to metformin | - | - | - | - | - | - |
| Unlikely | - | 0 (0) | 1 (33) | - | 1 (25) | - |
| Not related | - | 1 (100) | 2 (67) | - | 3 (75) | - |
| - | - | - | - | - | - | - |
| Treatment limiting toxicity* | 2 (9) | 5 (24) | 4 (18) | - | 11 (12) | p=0.043 |

All values are n (%).
*Either a dose reduction, or a temporary or permanent stop of treatment. In the maraviroc and metformin combined arm, two events led to only a change in metformin and six led to a change in both drugs.

AE: adverse event; ART: antiretroviral therapy; MET: metformin; MVC: maraviroc; SAE: serious adverse event

## **SDC9: Table 5S.** **Change in CD4+ T-cell count**

|  | Maraviroc | Metformin | Maraviroc and metformin | No drug (reference category) |
| --- | --- | --- | --- | --- |
| Total randomised | 23 (100) | 21 (100) | 22 (100) | 24 (100) |
|  |  |  |  |  |
| From W0-W24 | 20 (87) | 19 (90) | 20 (91) | 22 (92) |
| Absolute change (cells/mm^3^) | 24 (-54, 102) p=0.539 | -14 (-79, 51) p=0.663 | 21 (-54, 96) p=0.576 | - |
|  |  |  |  |  |
| From W0-W48 | 20 (87) | 19 (90) | 19 (86) | 23 (96) |
| Absolute change (cells/mm^3^) | 19 (-61, 99) p=0.629 | 16 (-63, 95) p=0.692 | 89 (12, 166) p=0.024 | - |

##

## **SDC10: Table 6S. Change in CD8+ T-cell count**

|  | Maraviroc | Metformin | Maraviroc and metformin | No drug (reference category) |
| --- | --- | --- | --- | --- |
| Total randomised | 23 (100) | 21 (100) | 22 (100) | 24 (100) |
|  |  |  |  |  |
| From W0-W24 | 20 (87) | 19 (90) | 20 (91) | 22 (92) |
| Absolute change (cells/mm^3^) | 91 (-13, 194) p=0.084 | -2 (-76, 72) p=0.949 | 74 (-17, 165) p=0.111 | - |
|  |  |  |  |  |
| From W0-W48 | 20 (87) | 19 (90) | 19 (86) | 23 (96) |
| Absolute change (cells/mm^3^) | 62 (-51, 175) p=0.279 | 80 (-58, 217) p=0.253 | 128 (20, 236) p=0.021 | - |

**SDC11: Table 7S. Weight change by arm**

|  | Maraviroc | Metformin | Maraviroc and metformin | No drug (reference category) |
| --- | --- | --- | --- | --- |
| Total randomised | 23 (100) | 21 (100) | 22 (100) | 24 (100) |
|  |  |  |  |  |
| From W0-W24 | 20 (87) | 19 (90) | 20 (91) | 23 (96) |
| Absolute change (kg) | -1 (-3, 2) p=0.537 | -2 (-4, 0) p=0.077 | -1 (-3, 1) p=0.328 | - |
|  |  |  |  |  |
| From W0-W48 | 20 (87) | 19 (90) | 19 (86) | 22 (92) |
| Absolute change (kg) | 0 (-3, 3) p=0.782 | -1 (-3, 1) p=0.286 | 0 (-2, 3) p=0.773 | - |

**SDC12: Table 8S. Change in ALP**

|  | Maraviroc | Metformin | Maraviroc and metformin | No drug (reference category) |
| --- | --- | --- | --- | --- |
| Total randomised | 23 (100) | 21 (100) | 22 (100) | 24 (100) |
|  |  |  |  |  |
| From W0-W24 | 21 (91) | 19 (90) | 20 (91) | 24 (100) |
| Absolute change (U/L) | 3 (-3, 9) p=0.274 | -1 (-7, 4) p=0.608 | -4 (-11, 2) p=0.171 | - |
|  |  |  |  |  |
| From W0-W48 | 21 (91) | 20 (95) | 19 (86) | 24 (100) |
| Absolute change (U/L) | -4 (-12, 4) p=0.286 | -6 (-14, 2) p=0.146 | -13 (-21, -5) p=0.002 | - |

**SUPPLEMENTARY FIGURES**

**SDC13: Figure 1S.** **Scatter plot of W0 liver fat vs W48 liver fat: planned scan date participants only**

Footnote: The black dashed line is a y=x reference line. Points below this line represent participants who reduced their liver fat; points above this line represent participants who increased their liver fat.

**SDC14: Figure 2S. Plot of ALT over time**

**SDC15: Figure 3S. Plot of AST over time**

**SDC16: Figure 4S. Plot of GGT over time**

**SDC17: Figure 5S. Plot of ALP over time**

**SDC18: Figure 6S.** **Plot of CD4+ T-cells over time**

**SDC19: Figure 7S. Plot of CD8+ T-cells over time**

**
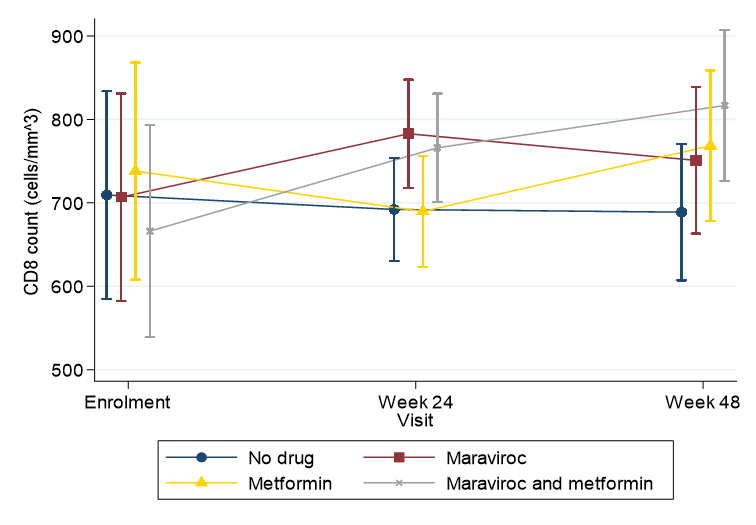
**

**SDC20: Figure 8S.** **Weight change by arm**

**SDC21: RADIOLOGY PROTOCOL REFERENCES**

1. Tang An, Justin Tan, Mark Sun, Gavin Hamilton, Mark Bydder, Tanya Wolfson, et al. **Nonalcoholic fatty liver disease: MR imaging of liver proton density fat fraction to assess hepatic steatosis.** *Radiology. 2013 May*;**267**(2):422-31.
2. Heba ER, Desai A, Zand KA, Hamilton G, Wolfson T, Schlein AN, et al. **Accuracy and the effect of possible subject-based confounders of magnitude-based MRI for estimating hepatic proton density fat fraction in adults, using MR spectroscopy as reference**. *J Magn Reson Imaging. 2016 Feb*;**43**(2):398-406.
3. Zand KA, Shah A, Heba E, Wolfson T, Hamilton G, Lam J, et al. **Accuracy of multiecho magnitude-based MRI (M-MRI) for estimation of hepatic proton density fat fraction (PDFF) in children**. *J Magn Reson Imaging. 2015 No*v;**42**(5):1223-32.
4. Idilman IS, Keskin O, Elhan AH, Idilman R, Karcaaltincaba M. **Impact of sequential proton density fat fraction for quantification of hepatic steatosis in nonalcoholic fatty liver disease**. *Scand J Gastroenterol. 2014 May*;**49**(5):617-24.
5. Idilman IS, Tuzun A, Savas B, Elhan AH, Celik A, Idilman R, et al. **Quantification of liver, pancreas, kidney, and vertebral body MRI-PDFF in non-alcoholic fatty liver disease.** *Abdom Imaging. 2015 Aug*;**40**(6):1512-9.
6. Imajo K, Kessoku T, Honda Y, Tomeno W, Ogawa Y, Mawatari H, et al. **Magnetic Resonance Imaging More Accurately Classifies Steatosis and Fibrosis in Patients With Nonalcoholic Fatty Liver Disease Than Transient Elastography**. *Gastroenterology. 2016 Mar*;**150**(3):626-637.e7.
7. Park CC, Nguyen P, Hernandez C, Bettencourt R, Ramirez K, Fortney L, et al. **Magnetic Resonance Elastography vs Transient Elastography in Detection of Fibrosis and Noninvasive Measurement of Steatosis in Patients With Biopsy-Proven Nonalcoholic Fatty Liver Disease.** *Gastroenterology. 2017 Feb*;**152**(3):598-607.e2.
8. Schwimmer JB, Middleton MS, Behling C, Newton KP, Awai HI, Paiz MN, et al. **Magnetic resonance imaging and liver histology as biomarkers of hepatic steatosis in children with nonalcoholic fatty liver disease.** *Hepatology. 2015 Jun*;**61**(6):1887-95.
9. Bannas P, Hernando D, Motosugi U, Roldan A, Reeder SB. **Emerging quantitative MRI biomarkers of diffuse liver disease**. *Clin Liver Dis (Hoboken). 2015 Jan 20*;**4**(6):129-132
10. Idilman IS, Keskin O, Celik A, Savas B, Elhan AH, Idilman R, et al. **A comparison of liver fat content as determined by magnetic resonance imaging-proton density fat fraction and MRS versus liver histology in non-alcoholic fatty liver disease**. *Acta Radiol. 2016 Mar*;**57**(3):271-8.
11. Di Martino M, Pacifico L, Bezzi M, Di Miscio R, Sacconi B, Chiesa C, et al. **Comparison of magnetic resonance spectroscopy, proton density fat fraction and histological analysis in the quantification of liver steatosis in children and adolescents.** *World J Gastroenterol. 2016 Oct 21*;**22**(39):8812-8819.
12. Kukuk GM, Hittatiya K, Sprinkart AM, Eggers H, Gieseke J, Block W, et al. **Comparison between modified Dixon MRI techniques, MR spectroscopic relaxometry, and different histologic quantification methods in the assessment of hepatic steatosis.** *Eur Radiol. 2015 Oct*;**25**(10):2869-79.
13. Paparo F, Cenderello G, Revelli M, Bacigalupo L, Rutigliani M, Zefiro D, et al. **Diagnostic value of MRI proton density fat fraction for assessing liver steatosis in chronic viral C hepatitis**. *Biomed Res Int. 2015*;**2015**:758164.
14. Runge JH, Bohte AE, Verheij J, Terpstra V, Nederveen AJ, van Nieuwkerk KM, et al. **Comparison of interobserver agreement of magnetic resonance elastography with histopathological staging of liver fibrosis***. Abdom Imaging. 2014 Apr*;**39**(2):283-90. doi: 10.1007/s00261-013-0063-z.

**SDC22: MAVMET PROTOCOL (VERSION 2.0 22-Apr-2020)**
